# Hydroxycinnamic Acid Derivatives Obtained from a Commercial *Crataegus* Extract and from Authentic *Crataegus* spp.^§^

**DOI:** 10.3797/scipharm.1404-02

**Published:** 2014-08-21

**Authors:** Ulrich Kuczkowiak, Frank Petereit, Adolf Nahrstedt

**Affiliations:** Univ. of Münster, Inst. of Pharm. Biology and Phytochemistry, Corrensstr. 48, D-48149 Münster, Germany.

**Keywords:** *Crataegus* spp., Rosaceae, Hydroxycinnamic acid derivatives, Hydroxycinnamoyl quinic acids, Hydroxycinnamoyl threonic acids

## Abstract

Eleven hydroxycinnamic acid derivatives were isolated from a 70% methanolic Crataegus extract (Crataegi folium cum flore) and partly verified and quantified for individual Crataegus species (C. laevigata, C. monogyna, C. nigra, C. pentagyna) by HPLC: 3-O-(E)-p-coumaroylquinic acid (1), 5-O-(E)-p-coumaroyl-quinic acid (2), 4-O-(E)-p-coumaroylquinic acid (3), 3-O-(E)-caffeoylquinic acid (4), 4-O-(E)-caffeoylquinic acid (5), 5-O-(E)-caffeoylquinic acid (6), 3,5-di-O-(E)-caffeoylquinic acid (7), 4,5-di-O-(E)-caffeoylquinic acid (8), (-)-2-O-(E)-caffeoyl-L-threonic acid (9), (-)-4-O-(E)-caffeoyl-L-threonic acid (10), and (-)-4-O-(E)-p-coumaroyl-L-threonic acid (11). Further, (-)-2-O-(E)-caffeoyl-D-malic acid (12) was isolated from C. submollis and also identified for C. pentagyna and C. nigra by co-chromatography. The isolates 10 and 11 were not found in the authentic fresh specimen, indicating that they may be formed during extraction by acyl migration from the 2-O-acylderivatives. Also, 9 and 11 are described here for the first time. All structures were assigned on the basis of their spectroscopic data (^1^H-, ^13^C-NMR, MS, optical rotation).

## Introduction

The monograph “hawthorn leaf and flower” (Crataegi folium cum flore) of the European Pharmacopoeia (PhEur) consists of the dried flowers and bearing branches of *Crataegus monogyna* Jacq. (Lindm.), *C. laevigata* (Poiret) D.C. (syn. *C. oxyacantha* Thuill.), *C. pentagyna* Waldst. et Kit. ex Willd., *C. nigra* Waldst. et Kit., and *C. azarolus* L. [1]. Preparations based on the hydroalcoholic extracts are commonly used as rational phyto-medicines for the treatment of cardiac insufficiency corresponding to class II (NYHA I-II). As for the pharmacological properties, phytochemical studies of the last three decades have been focused on flavonoids and oligomeric procyanidins [see ref. 2-6 and literature cited therein]. In contrast to the polyphenols, the knowledge about hydroxycinnamic acids and their derivatives in the drug material was limited. Some common cinnamic acids and benzoic acids were found after acidic hydrolysis of a methanolic total extract from the callus culture of *Crataegus monogyna* [7]. Besides some reports on chlorogenic acid (5-caffeoylquinic acid), the *N, N’, N”*-tricoumaroylspermidine has been unambiguously identified from hawthorn flowers (Crataegi flos) [7–12]. More recently, 5-*p*-coumaroylquinic acid was also detected from *Crataegus monogyna* cell suspension cultures [13]. The aim of the present study was to analyse the hydroxycinnamic acid derivatives (HCAs) from a commercial *Crataegus* extract (LI 132) in detail and to analyze them in the single *Crataegus* species used in the PhEur monograph.

## Results and Discussion

**Fig. 1.**
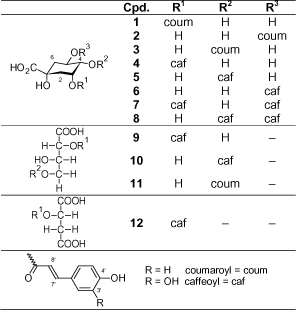
Structural formulas of the isolated compounds **1–12**

A range of hydroxycinnamic acid derivatives (HCAs) were isolated using a combination of CC on Sephadex LH-20 and different HPLC techniques (for formulas, see Fig. 1). The individual HCAs showed typical UV absorption bands for caffeic acids (**4–10**, **12**) at 240 and 336 nm or at 240 and 320 nm for *p*-coumaric acid derivatives (**1–3, 11**). The H-NMR spectra showed the typical aromatic AMX-spin system for caffeic and the AA‘XX’-spinsystem for *p*-coumaric acid moieties, respectively. All hydroxycinnamic acid derivatives showed signals for the protons of an (*E*)-configurated olefinic double bond with the typical coupling constant of about 16 Hz. Acylation positions at the HCA’s moieties were determined by ^1^H-/^13^C-NMR experiments according to the acylation shift of the esterfied position and chemical shift of geminal protons compared with the free quinic acid or aldonic acids, respectively [14]; they were also directly proven by HMBC experiments. The ^1^H-NMR data of the quinic acid derivatives were also confirmed by spectral simulation. Here we present the complete ^1^H- and ^13^C-NMR data sets in MeOH-*d_4_* of isomeric quinic acids in Tables 1S–4S in “Supplemental Information.” Concerning the problem of solvent effects in the structure dereplication of caffeoyl quinic acid, see [15].

The ESI-MS of **10** showed a m/z of 299 (M+H^+^) which differs clearly from those of quinic acid derivatives. The ^1^H-NMR (200 MHz) in methanol-*d_4_* revealed, in addition to the protons of the caffeoyl moiety, a broad signal at δ 4.2‱4.3 ppm, which was poorly separated at 600 MHz into the expected corresponding protons of the tertiary carbons (δ 4.24 ppm) and the two protons of the secondary carbon at δ 4.26 ppm (Table 5S). The ^13^C-NMR of **10** (D2O) showed two tertiary carbons at δ 71.9 and 71.7 ppm, a secondary at δ 65.7 ppm, and a carboxylic carbon at δ 176.5 ppm. GC-MS of the TMS-ether of **10** showed an m/z = 658 and m/z 439 and 219 as a result of an alpha splitting between the carbons C-2 and C-3, thereby confirming the trihydroxybutyric acid structure (Table 6S). Substitution of the acyl residue was confirmed at C-4 by HMBC experiments. NMR data and the optical rotation value of **10** were in agreement with the literature data [16, 17]. After hydrolysis of **10** and purification on polyamide, the [*α*]_*D*_^20^ value (+23.1, *c*1.6, water) corresponded to a sample of commercially available L-threonic acid. Thus, **10** was identified as (-)-4-*O*-(*E*)-caffeoyl–L-threonic acid.

The ESI-MS of **11** showed a relative molecular mass of m/z 282, the HR-ESI-MS of [M + H]^+^ = 282.0814 (calc. 282.0812 for C_13_H_15_O_7_). The AA’ XX’ spinsystem and the UV spectrum revealed a *p*-coumaric acid derivative. Loss of m/z 16 in comparison to **10** and **9** indicated an analog trihydroxybutyric acid derivative with a *p*-coumaroyl instead of a caffeoyl moiety. All signals of the trihydroxy butyric acid moiety were assigned by HMBC experiments. After hydrolysis of **11** and isolation of the products on polyamide, the [*α*]_*D*_^20^ was in good agreement to the corresponding commercially available L-threonic acid. Thus, **11** was identified as (-)-4-*O*-(*E*)-*p*-coumaroyl–L-threonic acid; its NMR data are shown in Table 1 and 5S.

The ^13^C- and ^1^H-NMR data and the optical rotation value of compound **9** coincided with the data obtained for 2-*O*-(*E*)-caffeoyl-L-threonic acid lactone [16]. However, in contrast to [16], the ESI–MS gave a relative molecular mass of 298 indicating a free caffeoyl-threonic acid. Additionally, the small ^3^J_H-2/3_-coupling constant of 2.4 Hz was inconsistent with a fixed *trans*-arrangement of the free hydroxyl functions caused by lactonisation of threonic acid [18]. These results required a new assignment of the threonic acid moiety by HMBC experiments and a comparison of the NMR data with those of free L-threonic acid and the synthesized L-threonic acid 1,4-lactone. Compound **9** showed no ^3^JH-4/C-1-coupling as a result of lactonisation, whereas the ^3^J_H-2/C-9’_-coupling and thus the point of attachment of the acyl moiety was clear. On careful inspection of the ^1^H-NMR data of free L-threonic acid (for data, see Table 7S), the acylation shift of the H-2 from δ 4.19 ppm to 5.28 ppm in **9** was obvious, but no similar shift for H-4 (δ 3.66, 3.61 ppm) for threonic acid or δ 3,63, 3,61 ppm for **9** was observed. In contrast, L-threonic acid lactone showed a clear acylation shift of the H-4 protons to δ 3.93 and 4.41 ppm (Table 1 and Table 7S). These results unambiguously assign the structure of compound **9** as (-)-2-*O*-(*E*)-caffeoyl-L-threonic acid. To the best of our knowledge, compounds **9** and **11** were described here for the first time.

**Tab. 1.** ^1^H- and ^13^C-NMR data of the new hydroxycinnamoyl aldonic acid derivatives **9** and **11** (in CD3OD = δ 3.30 ppm: M = multiplicity)

	9			11			9	11
H/C	(ppm)	*J*(Hz)	M	(ppm)	*J*(Hz)	M	(ppm)	(ppm)
1							172.2	175.8
2	5.28	2.4	*d*	4.25	2.0	*d*	73.6	72.3
3	4.20	2.4, 5.0, 9.0	*ddd*	4.22	2.0, 6.0, 8.0	*ddd*	72.7	72.2
4a	3.63	6.0, -11.0	*dd*	4.27	8.0, -11.0	*dd*	63.1	65.9
4b	3.61	8.0, -11.0	*dd*	4.26	6.0, -11.0	*dd*		
1’							127.8	127.2
2’	7.08	2.1	*d*	7.46	8.5, 2.3	*dd*	115.3	131.2
3’				6.80	8.5, 2.3	*dd*	146.8	116.8
4’							149.7	161.2
5’	6.79	8.2	*d*	6.80	8.5, 2.3	*dd*	116.5	116.8
6’	6.79	2.1, 8.2	*dd*	7.46	8.5, 2.3	*dd*	123.1	131.2
7’	7.68	15.8	*d*	7.64	16.0	*d*	147.9	146.8
8’	6.37	15.8	*d*	6.35	16.0	*d*	114.4	115.0
9’							168.5	169.0

Leaves with flowers of the following species were investigated (n= number of different origins): *C. monogyna* (n=3); *C. laevigata* (n=2), *C. nigra* (n=2), *C. pentagyna* (n=1). Only leaves, but no flowers of *C. azarolus* could be obtained. Quantitative analysis of the respective extracts by HPLC gave about 2.55% (m/m) of caffeic acid derivatives and 0.6% of *p*-coumaric acid derivatives. The variation of caffeic acid derivatives ranged from 0.85% for *C. laevigata* purchased from Tharandt, up to 3.12% for *C. monogyna* from Marburg. The variation of *p*-coumaric acid derivatives ranged from 0.05% for *C. laevigata* (Marburg) to 0.32% for *C. monogyna* (Marburg) (see Tables 2 and 3).

Compounds **10** and **11** isolated from the LI 132 extract were not detected in the authentic fresh *Crataegus* species. Because the isolation procedure from fresh material and the LI 132 extract was identical, their presence in the LI 132 extract may be explained by the partial acyl shift during the extraction of the drug material of the 2-*O*-acylderivatives as shown for *Chelidonium majus* [16]. Compound **8** was found in lower quantities in the herbal material than in the extract, which may again indicate an isomerisation from compound **7**.

**Tab. 2.** Amount of hydroxycinnamic acid derivatives in hawthorn leaves with flowers (Crataegi folium cum flore) in [m/m %] and ± relative standard deviation (n=3)

Comp.	*Crataegus monogyna* Münster 50% flowers	*Crataegus monogyna* Marburg 81% flowers	*Crataegus monogyna* Tharandt 50% flowers	*Crataegus laevigata* Tharandt 53% flowers	*Crataegus laevigata* Marburg 80% flowers
**4**	0.25%±1.4%	1.15%±0.6%	0.54%±1.0%	-	-
**5**	**—**	0.04%±7.8%	0.06%±1.2%	0.09%±0.8%	0.27%±3.5%
**6**	1.01%±1.8%	1.39%±1.1%	0.80%±1.1%	0.71%±9.2%	0.57%±0.3%
**1**	0.05%±2.1%	0.13%±1.6%	0.10%±1.3%	**—**	**—**
**3**	**—**	**—**	0.01%±4.9%	0.02%±7.2%	0.02%±5.3%
**2**	0.11%±1.2%	0.08%±1.9%	0.07%±1.0%	0.05%±11%	0.03%±2.1%
**7**	0.23%±9.0%	0.26%4.6%	0.05%±16%	0.06%±14%	0.12%±7.0%
**8**	<0.01%	**—**	**—**	**—**	**—**
**9**	0.09%±1.8%	0.29%+1.4%	0.18%±4.4%	-	-
**10**	-	-	-	-	-
**12**	-	-	-	-	-
caffeic acid derivatives	1.58%±2.6%	3.12%±1.3%	1.63%±1.9%	0.85%±8.8%	0.95%±3.7%
*p*-coumaric acid derivatives	0.31%±1.7%	0.32%±1.6%	0.22%±1.2%	0.08%±9.8%	0.05%±2.9%

**Tab. 3.** Amount of hydroxycinnamic acid derivatives in hawthorn leaves with flowers (Crataegi folium cum flore) in [m/m %] and ± relative standard deviation (n=3)

Comp.	*Crataegus nigra* Marburg 78% flowers	*Crataegus nigra* Münster 42% flowers	*Crataegus pentagyna* Tharandt 67% flowers	LI 132 97020100	LI 132 96970100
**4**	1.43%±1.4%	0.93%±3.0%	0.77%±1.0%	0.33%±0.7%	0.49%±0.2%
**5**	0.17%±1.4%	0.02%±12%	0.14%±1.3%	0.30%±0.9%	0.48%±0.3%
**6**	0.38%±0.6%	1.22%±1.5%	0.55%±0.4%	1.30%±0.9%	1.56%±0.3%
**1**	0.09%±2.5%	0.07%±2.6%	0.05%±1.9%	0.14%±2.7%	0.15%±0.1%
**3**	**—**	**—**	0.03%±4.3%	0.14%±0.9%	0.17%±0.2%
**2**	**—**	0.07%±4.1%	0.02%±3.2%	0.15%±1.3%	0.15%±1.4%
**7**	0.12%±5.0%	0.22%±2.6%	0.08%±3.1%	0.10%±5.3%	0.17%±17%
**8**	**—**	**—**	**—**	0.11%±2.5%	0.15%±6.9%
**9**	0.56%±1.8%	0.58%±0.4%	0.36%±0.8%	0.09%±1.1%	0.13%±2.0%
**12**	0.13%±4.8%	**—**	0.06%±4.8%	**—**	**—**
**10**	**—**	**—**	**—**	0.15%±1.8%	0.24%±0.6%
**11**	—	—	—	0.09%±0.3%	0.11%±0.4%
caffeic acid derivatives	2.81%±1.7%	2.6%±1.9%	1.96%±1.0%	2.27%±1.1%	3.08%±1.2%
*p*-coumaric acid derivatives	0.29%±4.3%	0.16%±2.9%	0.21%±2.7%	0.56%±1.4%	0.64%±0.7%

Compound **12**, which was isolated from *C. submollis,* was verified for *C. pentagyna* and origins of *C. nigra* by HPLC. The other species showed a very similar qualitative pattern of hydroxycinnamic acid derivatives. Only for the samples of *C. laevigata,* the 3-*O*-mono-hydroxycinnamic quinic acids and the aldonic acid derivatives were not observed.

Further investigations of the flowers and leaves of the different species separately and in more detail showed that the 3-*O*-hydroxycinnamic quinic acids were only detectable in flowers (data not shown). The contribution of the HCA derivatives of *Crataegus* to cardiac diseases is to the best of our knowledge not known. However, a recent systematic review and meta-analysis on chlorogenic acid and its derivatives, mainly from green coffee beans, concluded that evidence suggests that chlorogenic acid intake causes a statistically significant reduction in systolic and diastolic blood pressures [19 and references cited therein]. In view of the relatively high amount of HCAs in *Crataegus* crude drugs of c. 1% to more than 3%, it seems worthwhile to investigate whether they participate in the therapeutical efficacy of *Crataegus* preparations.

## Experimental

### General

Optical rotations were measured on a Perkin-Elmer 241 apparatus, mass spectra on a Finnigan LC-Q and Quattro LC Z (ESI-MS), and on a Finnigan MAT 8230 combined with a Varian 3400 GC using an HP-5 column (25 m) (GC-MS). The mass spectra were calibrated using a reference spectrum of sodium formate. The exact mass for **11** was determined with a Bruker micrOTOF-qII mass spectrometer coupled to a Dionex Ultimate 3000 RS UHPLC System. Separation was achieved over an RP18 column (Dionex Acclaim 120 C18, 2.2 µm, 2.1 x 100 mm) eluted by a binary water-acetonitrile gradient containing 0.1% formic acid at 0.8 mL/min, +10% acetonitrile/min beginning at 5% acetonitrile; the mass spectrum was acquired in positive mode over a m/z range of 50–1500 with a nebulizer pressure of 4.0 bar (N2), dry gas flow of 9.0 L/min at 220°C (N2), capillary voltage of 4500 V and 8 eV collision energy. ^1^H- and ^13^C-NMR data were recorded at 200 and 50 MHz, respectively, with a Varian Gemini 200 spectrometer or at 600 MHz and 150 MHz with a Varian Unity 600 (MeOH or DMSO as internal standard). Offline data processing and ^1^H-NMR simulation was done with the Nuts programme package (Acorn NMR, Vermont CA).

Analytical TLC was carried out on silica gel aluminum plates (0.2 mm, Merck) using ethyl acetate/formic acid/water (90:5:5), ethyl acetate/methanol/water/formic acid (100:4:8:5), and ethyl acetate/formic acid/acetic acid/water (100:11:11:27). Spots were visualized with Naturstoff reagent (1% MeOH) or 1% ethanolic FeCl_3_ solution.

The analytical HPLC (system 1) was performed with a Waters 600 controller, Waters 990 PAD; software: millennium; column: Prosep C18 (Latek), 5 µm, and 2 mL/min. flow rate. Mobile phase A: acetonitrile, B: 0.1% TFA. Elution gradient: 0–20 min. 0% A 𠆒8% A, 20–35 min. 8% A→ 21% A, 35–40 min. 21% A→ 40% A, 40–45 min. 40% A→ 100 % A, 45–60 min. 100% A→ 0% A.

The semi-preparative HPLC (system 2) was carried out with a Waters 600 controller, Waters 515 pump, and Waters Lambda-Max 481 LC spectrophotometer on the Hypersil ODS (Knauer, Germany), 5 µm, 250 × 16 mm, or preparative (system 3) on the Eurospher 100-C18 (Knauer, Germany, 7 µm, 250 × 20 mm).

The quantitative HPLC (system 4) was performed with a Waters 600 controller, Waters 996 PAD; software: Millennium; column: Discovery C18, 5 µm, 150 × 4.6 mm and 2 mL/min. flow rate. Mobile phase A: acetonitrile, B: methanol C: 0.1% TFA. Elution gradient: 0–12 min. 5% A →12% A, 0 % B, 95% C→ 88% C; 12–20 min. 12% A→ 15% A, 0% B→ 15% B, 88% C → 70% C; 20–30 min. 15% A→ 23% A, 15% B, 70% C→ 62% C; 30–35 min. 23% A→ 5% A, 15% B→ 0% B, 62% C→ 95% C.

### Extract and Plant Material

The 70% methanolic *Crataegus* extract LI 132 (Ch.-Nr. 96970100 and 97020100) was obtained from Lichtwer AG, Berlin. It was standardized on 2% flavonoids calculated as hyperoside by the method of DAB 10 [20]. The drug-extract-ratio was 1 : 5.5.

Hawthorn leaves with flowers were harvested in 1999 at different botanical gardens in Germany (Tharandt, Marburg, Münster) and the Experimental Garden of our institute in Münster. Leaves from *C. submollis* stem from the Botanical Garden in Münster. All collected plant material was air dried at 25°C. Voucher specimen are deposited at the Institute under No. 143-147, 149-151).

### Qualitative and Quantitative Determination of Hydroxycinnamic Acid Derivatives

An amount of 600 mg of powdered herbal material was extracted three times with 25 mL MeOH/water (7:3). After centrifugation, the supernatants were combined and concentrated to less than 10 mL and finally adjusted with H2O to 20.0 mL and kept for 24h at 4°C. After filtration of the supernatant, aliquots were used for HPLC (system 4). The LI 132 extract was dissolved in 20.0 mL water, filtered, and used for HPLC (system 4). Chlorogenic acid (for monocaffeoyl-derivatives), cynarin (for dicaffeoyl-derivatives), and *p*-coumaric acid (for monocoumaroyl-derivatives) were used as external standards for calibration. The correlation coefficient of the standard curves were r^2^= 0.9999. Correction factors for all isolated structures were determined by injection of 7 pmol of monohydroxycinnamic acid derivatives and 4 pmol of dicaffeoylquinic acid derivatives. Correction factors were: 1.27 (**1**), 1.07 (**2**), 1.10 (**3**), 1.23 (**4**), 1.21 (**5**), 1.26 (**6**), 1.45 (**7**), 1.31 (**8**), 1.65 (**10**), 1.36 (**11**), and 1.45 (**12**).

### Isolation Procedure

An amount of 350 g of a commercial 70% methanolic extract *(Crataegus* LI-132) was suspended in water (700 mL) and stored for 24 h at 4°C to precipitate chlorophyll. After filtration, the remaining chlorophyll was removed by shaking with dichloromethane. The purified aqueous phase was acidified with HCl at pH 2 and successively extracted with 3 x 700 mL diethyl ether (A), 5 x 700 mL ethyl acetate (B), and 3 x 700 mL *n*-butanol (C). After removal of the solvents, the extracts were lyophilized to yield 3.1 g for A, 21.7 g for B, and 50.0 g for C. An aliquot (19.6 g) of the EtOAc-extract (B) enriched with phenolics was further fractionated by column chromatography over Sephadex LH-20 (510 x 55 mm) with 11.2 L 50% methanol to give 11 subfractions (I-XI), and the first 570 mL were discarded. Fractions were monitored by TLC on silica gel and HPLC on RP-18 material (system 1). A portion (320 mg) of Sephadex-fraction III (elution volume 1350–1490 mL, 1.25 g) was purified with the semi-preparative HPLC (system 2) to yield **1** (38 mg) and **4** (38 mg). A part (480 mg) of Sephadex-fraction IV (elution volume 1491–1630 mL, 2.3 g) was fractionated by the semi-prep. The HPLC yielded pure **3** (9 mg), **2** (47 mg), and **5** (40 mg). Compound **9** (36 mg) was obtained after fractionation of a portion (650 mg) of Sephadex-fraction V (elution volume 1631–1770 mL, 1.28 g) by the same semi-prep. HPLC system as stated above. Compounds **6** (160 mg) and **11** (19 mg) were achieved after the semi-prep. HPLC of a portion (650 mg) of the Sephadex-fraction VI (elution volume 1771–2130 mL, 1.52 g). Compound **10** (16 mg) was obtained after the purification of a part (300 mg) of Sephadex-fraction VII (elution volume 2131–2500 mL, 1.7 g) by the HPLC (system 2). Further purification by the prep. HPLC (system 3) of a portion (250 mg) of Sephadex-fraction X (elution volume 5991–8420 mL, 0.51 g) yielded **7** (12 mg) and **8** (6 mg). Compound **12** was obtained from 240 g of the air dried leaves of *C. submollis* by extraction with 70% methanol. After removal of methanol, the remaining aqueous phase was acidified (pH 2) with HCl and further extracted with ethyl acetate, concentrated to a brown-yellow coloured extract (13.3 g). A portion (7.2 g) was fractionated (CC) over Sephadex LH-20 (400 x 25 mm) with 50% methanol. A part (270 mg) of fraction 20 (elution volume: 400–500 mL, 1.3 g) was purified by the prep. HPLC (system 3) to yield pure **12** (65 mg).

### Compounds

#### 3-O-(E)-p-Coumaroylquinic acid (1)

[*α*]^2^_*D*_^0^ -2.1° (*c* 0.36, methanol); ESI-MS (m/z): 337.4 [M - H]^-^ R_t_: 7.9 min (system 4); ^1^H]^-^ and ^13^C-NMR data see Tables 1S and 3S in “Supporting Information.”

#### 5-O-(E)-p-Coumaroylquinic acid (2)

[*α*]^2^_*D*_^0^ -42.5° (*c* 0.36, methanol); ESI-MS (m/z): 337.4 [M - H]^-^; R_t_: 12.9 min (system 4); ^1^H- and ^13^C-NMR data see Tables 1S and 3S in “Supporting Information.”

#### 4-O-(E)-p-Coumaroylquinic acid (3)

[*α*]^2^_*D*_^0^ -70.8° (*c* 0.36, methanol); ESI-MS (m/z): 337.4 [M - H]^-^; ESI-MS/MS (m/z) (rel. int. in %) (337.4): 173 [M – 164 - H]^-^ (73); 119 [M – 218 - H]^-^ (71); 93 [M – 218 - H]^-^ (100); R_t_: 13.9 min (system 4); ^1^H- and ^13^C-NMR data see Tables 1S and 3S in “Supporting Information.”

#### 3-O-(E)-Caffeoylquinic acid (4)

[*α*]^2^_*D*_^0^ +7° (*c* 0.1, methanol); ESI-MS (m/z): 353.4 [M - H]^-^; LC/MS (m/z) (rel. int. in %): 371.8 [M + NH_4_]^+^ (100); 725.7 [2xM + NH_4_]^+^ (18); 355 [M + NH_4_]^+^ (27); R_t_: 5.4 min (system 4); ^1^H- and ^13^C-NMR data see Tables 2S and 3S in “Supporting Information.”

#### 4-O-(E)-Caffeoylquinic acid (5)

[*α*]^2^_*D*_^0^ -60.3° (*c* 0.1, methanol); ESI-MS (m/z): 353.4 [M - H]^-^; R_t_: 10.7 min (system 4); ^1^H- and ^13^C-NMR-data see Tables 2S and 3S in “Supporting Information.”

#### 5-0-(E)-Caffeoylquinic acid (6)

[α]_D_^20^ -34° (*c* 0.1, methanol); LC/MS (m/z) (rel.int. in %): 467.2 [M + TFA - H]^-^ (100); 820,6 [2x M + TFA - H]^-^ (4); R_t_: 9 min (system 4); ^1^H- and ^13^C-NMR data see Tables 2S and 3S in “Supporting Information.”

#### 3,5-Di-0-(E)-Caffeoylquinic acid (7)

[α]_D_^20^ -139° (*c* 0.12, methanol); LC/MS (m/z) (rel.int. in %): 628.9 [M + TFA - H]^-^ (100); R_t_: 22.2 min (system 4); ^1^H- and ^13^C-NMR data see Table 4S in “Supporting Information.”

#### 4, 5-Di-0-(E)-Caffeoylquinic acid (8)

[α]_D_^20^ -170° (c 0.12, methanol); LC/MS (m/z) (rel.int. in %): 628.9 [M + TFA - H]^-^ (100); R_t_: 24.6 min (system 4); ^1^H- and ^13^C-NMR data see Table 4S in “Supporting Information.”

#### (-)-2-0-(E)-Caffeoyl-L-threonic acid (9)

[α]_D_^20^ -17.6° (c 0.13, water); ESI-MS (m/z) (rel. int. in %): 297 [M - H]^-^ (100); 279 [M - 18 - H]^-^ (4); 135 [threonic acid - H]^-^; 117 [threonic acid - H2O]”; ESI- MS/MS (m/z) (rel. int. in %) (297): 179 [caffeoyl - H]^-^ (10); 135 [threonic acid - H]” (100); ESI-MS/MS/MS (m/z) (rel. int. in %) (135): 117 [threonic acid - H_2_O]^-^ - H]^-^ (100); 75 (13) (a-splitting of m/e 117); Rt: 8.3 min (system 4); ^1^H- and ^13^C-NMR data see Table 1 and Tables 5S, 6S in “Supporting Information.”

#### (-)-4-0-(E)-Caffeoyl-L-threonic acid (10)

[α]_D_^20^ -18.8° (*c* 0.14, water); -24.7 (*c* 0.35, methanol); ESI-MS (m/z) (rel. int. in %): 297 [M - H]^-^ (100); 319 [M + Na - 2H]^-^ (90); ESI-MS/MS (m/z) (rel. int. in %) (297): 179 [caffeoyl - H]^-^ (7), 135 [threonic acid - H]^-^ (100); GC-MS of the silylated compound: GC-MS (m/z) (rel. int. in %): 658 (32); 643 (12); 439 (92); 307 (100); 219 (18); Rt: 12.5 min (system 4); ^1^H- and ^13^C-NMR data see Tables 5S and 6S in “Supporting Information.”

#### (-)-4-0-(E)-p-Coumaroyl-L-threonic acid (11)

[α]_D_^20^ -34° (c 0.1, methanol); ESI-MS (m/z): 281 [M - H]^-^; 563 [2M - H]^-^; 305 [M + Na]^+^; 587 [2M + Na]^+^; HR-ESI-MS (m/z): 283.0814 [M + H]^+^ (calc. 283.0812 for C13H15O17); Rt: 16.4 min (system 4); ^1^H- and C-NMR data see Table 1 and Tables 5S, 6S in “Supporting Information.”

#### (-)-2-0-(E)-Caffeoyl-D-malic acid (12)

from *Crataegus submollis:* [α]_D_^20^ -27.9° (c 0.24, methanol); ESI-MS (m/z):281 [M - H]^-^; 563 [2M - H]^-^; 305 [M + Na]^+^; 587 [2M + Na]^+^; Rt: 15.8 min (system 4); ^1^H- and ^13^C-NMR data see Tables 5S and 6S in “Supporting Information.”

### Hydrolysis of 10 and 11 and Product Analysis

The hydrolysis and analysis of free L-threonic acid were performed according to Hahn [16]. Amounts of 35 mg of **11** and 10 mg of **10** were hydrolyzed enzymatically (AB enzymes EL-1/77 “Röhm-Enzym”; pH 5, 37°C, 72 h) and the optical rotation was measured after purification on polyamide (SC-6, Macherey & Nagel, Düren; 220 x 5 mm) and checked for purity by TLC. The L-threonic acid thus obtained from **11** and **10** showed an optical rotation value of [*α*]_*D*_^20^ +18.4° (*c* 0.16, water) and [*α*]_*D*_^20^ +23.1° (*c* 0.16, water), respectively. L-Threonic acid was prepared from its hemicalcium salt × 1 H_2_O (Sigma-Aldrich, > 97%) using cation exchange on DOWEX 50 WX 8 (Serva, Heidelberg) and purified in the same way showed [*α*]^20^_*D*_ +31.1° (*c* 0.93, water).

### Synthesis of γ-L-Threonic Acid Lactone

To demonstrate a missing acylation shift of **9** at position 4 (see discussion), γ-L-threonic acid lactone was synthesized from L-threonic acid lactone hemicalcium salt x 1 H_2_O (Sigma-Aldrich) by a modified method according to Angelotti [21]. After converting to L-threonic acid (see above), the solution was concentrated and dried over P_2_O_5_ under vacuum for five days. The resulting ^1^H- and ^13^C-NMR spectra were compared with the data of **11** and free L-threonic acid lactone (Table 1 and 7S).

## Notes

Supporting Information Tables 1S–7S containing all ^1^H- and ^13^C-NMR data of the isolated compounds **1–12** and those of L-threonic acid and γ-L-threonic acid lactone are available in the online version (Type: PDF, Size: ca. 0.1 MB): http://dx.doi.org/10.3797/scipharm.1404-02.
