# Supplementary material for: Hydroxycinnamic Acid Derivatives Obtained from a Commercial Crataegus Extract and from Authentic Crataegus spp
Source: Sci Pharm. 2014 Aug 21;82(4):835–46. doi: 10.3797/scipharm.1404-02 (PMC4475804; doi:10.3797/scipharm.1404-02)
Supplement: Supporting Information — Tables 1S–7S containing all 1H- and 13C-NMR data of the isolated compounds 1–12 and those of L-threonic acid and γ-L-threonic acid lactone are available in the online version (Type: PDF, Size: ca. 0.1 MB): http://dx.doi.org/10.3797/scipharm.1404-02. [file Scipharm-2014-82-835-Support.pdf]

Scientia Pharmaceutica www.scipharm.at

Open Access

Supporting Information to

Hydroxycinnamic Acid Derivatives Obtained from a Commercial *Crataegus* Extract and from Authentic *Crataegus* spp.

Ulrich KUCZKOWIAK, Frank PETEREIT, Adolf NAHRSTEDT

Published in Sci Pharm. 2014; 82: 835–846 doi:10.3797/scipharm.1404-02

Available from: http://dx.doi.org/10.3797/scipharm.1404-02

© Kuczkowiak *et al.*; licensee Österreichische Apotheker-Verlagsgesellschaft m. b. H., Vienna, Austria.

This is an Open Access article distributed under the terms of the Creative Commons Attribution License (http://creativecommons.org/licenses/by/3.0/), which permits unrestricted use, distribution, and reproduction in any medium, provided the original work is properly cited.

Table of Contents

Tab. 1S. ^{1}H-NMR data of monocoumaroylquinic acids, *p*-coumaric acid, and free D-(−)-quinic acid
Tab. 2S. ^{1}H-NMR data of monohydroxycinnamoylquinic acids, caffeic acid, and free D-(−)-quinic acid
Tab. 3S. ^{13}C-NMR data of monocaffeoylquinic acids and free D-(−)-quinic acid
Tab. 4S. ^{1}H-NMR and ^{13}C-NMR data of dicaffeoylquinic acids
Tab. 5S. ^{1}H-NMR data of hydroxycinnamoyl aldonic and malic acid derivatives
Tab. 6S. ^{13}C-NMR data of hydroxycinnamoyl aldonic and malic acid derivatives
Tab. 7S. ^{1}H-NMR of L-threonic acid and γ-L-threonic acid lactone

Tab. 1S. ^{1}H-NMR data of monocoumaroylquinic acids, *p*-coumaric acid, and free D-(−)-quinic acid (rel. to CD_3OD = δ 3.30 ppm; M = multiplicity)

	**1**		**3**		4			D-(−)-quinic acid	
H	(ppm)	*J* (Hz)	(ppm)	*J* (Hz)	(ppm)	*J* (Hz)	M	(ppm)	*J* (Hz)
2ax	2.20	2.4; -14	2.17	2.8; -13.5	2.17	3.4; -14.2	*dd*	2.06	3.3; -14.5
2eq	2.14	2.0; 4.3; -14	2.07	2.4; 3.8; -13.5	2.04	2.2; 5.3; -14.2	*ddd*	2.02	2.0; 3.3; -14.5
6ax	1.95	10; -13	2.01	11.0; -13.5	2.08	9.3; -13.5	*dd*	1.85	11.0; -13.5
6eq	2.14	2.0; 4.3. -13	2.20	2.4; 4.0; -13.5	2.22	2.2; 4.4; -13.5	*ddd*	2.12	2.0; 4.7; -13.5
3	5.36	2.4; 3.2; 4.3	4.29	2.8; 3.3; 3.8	4.16	2.9; 3.4; 5.3	*ddd*	4.08	3.3; 3.7; 3.2
4	3.64	3.2; 9	4.80	3.3; 9.4	3.72	2.9; 8.4	*dd*	3.39	3.2; 9.2
5	4.15	9; 4.3; 10	4.28	4.0; 9.4; 11.0	5.33	4.4; 8.4; 9.3	*ddd*	3.99	4.7; 9.2; 11
								p-coumaric acid	
2'	7.45	2.3; 8.6	7.47	2.3; 8.6	7.46	2.3; 8.6	*d(d)*	7.41	2.3; 8.6
3'	6.80	2.3; 8.6	6.80	2.3; 8.6	6.80	2.3; 8.6	*d(d)*	6.79	2.3; 8.6
5'	6.80	2.3; 8.6	6.80	2.3; 8.6	6.80	2.3; 8.6	*d(d)*	6.79	2.3; 8.6
6'	7.45	2.3; 8.6	7.47	2.3; 8.6	7.46	2.3; 8.6	*d(d)*	7.41	2.3; 8.6
7'	7.65	15.9	7.70	15.9	7.62	15.9	*d*	7.59	15.9
8'	6.36	15.9	6.42	15.9	6.32	15.9	*d*	6.27	15.9
	400 MHz		600 MHz		400 MHz			200 MHz	

Tab. 2S. ^{1}H-NMR data of monohydroxycinnamoylquinic acids, caffeic acid, and free D-(−)-quinic acid (rel. to CD_3OD = δ 3.30 ppm; M = multiplicity)

	**4**		**5**		**6**			D-(−)-quinic acid	
H	(ppm)	*J* (Hz)	(ppm)	*J* (Hz)	(ppm)	*J* (Hz)	M	(ppm)	*J* (Hz)
2ax	2.18	3.2; -14.3	2.17	2.4; -14.4	2.17	3.4; -14.2	*dd*	2.05	3.3; -14.5
2eq	2.13	2.0; 4.3; -14.3	2.07	2.4; 3.4; -14.4	2.04	2.2; 5.4; -14.2	*ddd*	2.02	2.0; 3.3; -14.5
6ax	1.94	9.7; -13.4	2.01	10.5; -13.5	2.07	9.3; -13.5	*dd*	1.85	11.0; -13.5
6eq	2.11	2.0; 4.0; -13.4	2.21	2.4; 4; -13.5	2.22	2.2; 4.4; -13.5	*ddd*	2.12	2.0; 4.7; -13.5
3	5.34	3.2; 3.2; 4.3	4.28	2.4; 3.3; 3.4	4.16	3.3; 3.4; 5.4	*ddd*	4.08	3.3; 3.7; 3.4
4	3.63	3.2. 8.3	4.80	3.3; 9.6	3.72	3.3; 8.4	*dd*	3.39	3.4; 9.2
5	4.14	4.0; 8.3; 9.7	4.29	4.0; 9.6; 10.5	5.33	4.4; 8.4; 9.3	*ddd*	3.99	4.7; 9.2; 11
								caffeic acid	
2'	7.03	1.90	7.06	1.90	7.04	1.90	*d*	7.03	1.9
3'	–	–	–	–	–	–	*d*	–	
5'	6.76	8.2	6.78	8.2	6.77	8.0	*d*	6.77	8.2
6'	6.93	1.9; 8.2	6.95	1.9; 8.2	6.95	1.9; 8.0	*d*	6.92	1.9; 8.2
7'	7.57	15.9	7.63	16.0	7.55	16.1	*d*	7.53	15.9
8'	6.30	15.9	6.36	16.0	6.26	16.1	*d*	6.21	15.9
	200 MHz		400 MHz		360 MHz			200 MHz	

Tab. 3S. ^{13}C-NMR data of monocaffeoylquinic acids and free D-(−)-quinic acid (rel. to CD_3OD = δ 49 ppm, 50 MHz)

	**1**	**3**	**4**	**4**	**5**	**6**	D-(−)-quinic acid
C	(ppm)	(ppm)	(ppm)	(ppm)	(ppm)	(ppm)	(ppm)
1	75.42	76.61	76.22	75.46	76.61	76.17	76.88
2	36.75	38.41	38.26	36.77	38.43	38.12	38.43
3	73.03	69.67	71.36	73.02	69.69	71.35	71.93
4	74.87	79.34	73.58	74.84	79.34	73.57	77.1
5	68.29	65.52	72.02	68.34	65.54	71.86	68.03
6	41.57	42.72	38.88	41.53	42.74	38.85	42.39
7	178.29	177.27	177.04	178.5	177.27	176.96	177.56
1'	127.44	127.32	127.30	128.04	127.92	127.78	
2'	131.05	131.25	131.12	115.91	115.42	115.26	
3'	116.80	116.82	116.84	146.81	146.74	146.59	
4'	161.06	161.13	161.2	149.35	149.47	149.36	
5'	116.80	116.82	116.84	116.51	116.53	116.49	
6'	131.05	131.25	131.12	122.84	122.94	122.94	
7'	146.42	146.74	146.66	146.81	147.12	147.01	
8'	115.94	115.44	115.42	115.21	115.27	115.26	
9'	169.00	168.99	168.70	169.04	169.03	168.70	

Tab. 4S. ^{1}H-NMR and ^{13}C-NMR data of dicaffeoylquinic acids (in CD_3OD = δ 3.30 ppm and 49.0 ppm)

	**7**		**8**				**7**	**8**
H	(ppm)	*J* (Hz)	(ppm)	*J* (Hz)	M	C	(ppm)	(ppm)
2ax	2.31	4.0; -13.8	2.30	3; -14.2	*dd*	C-1	74.77	76.15
2eq	2.15	1.5; 7.2; -13.8	2.10	2; 5.2; -14.2	*ddd*	C-2	36.04	38.45
6ax	2.20	7.5; 14.0	2.23	9.3; -14	*dd*	C-3	72.58	69.00
6eq	2.23	1.5; 4.2; -14	2.28	2; 5; -14	*ddd*	C-4	70.71	75.86
3	5.41	3; 4.0; 7.2	4.36	3; 3; 5.2	*ddd*	C-5	72.13	69.42
4	3.98	3; 7.5	5.11	3; 9.3	*dd*	C-6	37.70	39.45
5	5.41	4.2; 7.5; 7.5	5.61	5; 9.3; 9.3	*ddd*	C-7	17.74	176.76
						C-1'/1"	127.99 / 127.87	127.78 / 127.72
						C-2'/2"	115.97 / 115.68	115.27 / 115.27
2'/2"	7.06 / 7.06	2.1	7.02 / 7.00	2.3	*d*	C-3'/3"	146.77 / 146.77	147.70 / 147.57
3'/3"	-	-	-	-	*d*	C-4'/4"	149.56 / 149.47	149.67 / 149.67
5'/5"	6.77 / 6.77	8.1	6.73 / 6.73	8.3	*d*	C-5'/5"	116.51 / 116.51	116.53 / 116.53
6'/6"	6.96 / 6.96	2.1; 8.1	6.93 / 6.89	2.3; 8.3	*dd*	C-6'/6"	123.01 / 122.94	123.10 / 123.10
7'/7"	7.61 / 7.57	16.1	7.60 / 7.51	16.1	*d*	C-7'/7"	147.03 / 147.26	146.77 / 146.77
8'/8"	6.34 / 6.26	16.1	6.28 / 6.18	16.1	*d*	C-8'/8"	115.33 / 115.20	114.78 / 114.84

Tab. 5S. ^{1}H-NMR data of hydroxycinnamoyl aldonic and malic acid derivatives (in CD_3OD = δ 3.30 ppm: M = multiplicity)

	10			11			9			12			
H	(ppm)	*J* (Hz)	M	(ppm)	*J* (Hz)	M	(ppm)	*J* (Hz)	M	H	(ppm)	*J* (Hz)	M
2	4.24	-	*d*	4.25	2.0	*d*	5.28	2.4	*d*	2	5.48	4.4; 7.8	*dd*
3	4.24	-	*ddd*	4.22	2.0; 6.0; 8.0	*ddd*	4.2	2.4; 5.0; 9.0	*ddd*	3a	2.96	-16; 4.4	*dd*
4a	4.26	-	*dd*	4.27	8; -11	*dd*	3.63	6.0; -11.0	*dd*	3b	2.91	-16; 7.8	*dd*
4b	4.26		*dd*	4.26	6; -11	*dd*	3.61	8.0; -11.0	*dd*				
2'	7.04	1.7	*d*	7.46	8.5; 2.3	*d*	7.08	2.1	*d*	2'	7.06	2.0	*d*
3'	-	-	-	6.80	8.5; 2.3	*dd*	-	-	-	3'	-	-	-
5'	6.77	8.1	*d*	6.80	8.5; 2.3	*dd*	6.79	8.2	*d*	5'	6.78	8.0	*d*
6'	6.95	1.7; 8.1	*dd*	7.46	8.5; 2.3	*dd*	6.97	2.1; 8.2	*dd*	6'	6.95	2.1; 8.0	*dd*
7'	7.58	15.9	*d*	7.64	16.0	*d*	7.68	15.8	*d*	7'	7.60	15.8	*d*
8'	6.29	15.9	*d*	6.35	16.0	*d*	6.37	15.8	*d*	8'	6.31	15.8	*d*
	600 MHz			600 MHz			200 MHz				200 MHz		

Tab. 6S. ^{13}C-NMR data of hydroxycinnamoyl aldonic and malic acid derivatives (50 MHz, in CD_3OD = δ 49.0 ppm; 10 also in D_2O)
* These assignments may be interchanged

	10	10	11	9	12
C	(ppm)	(ppm)	(ppm)	(ppm)	(ppm)
	-	176.5	175.8	172.2	173.0*
2	71.4	71.9	72.3	73.6	69.9
3	71.4	70.7	72.2	72.7	37.1
4	66.0	65.7	65.9	63.1	179.6*
1'	127.8	127.7	127.2	127.8	127.7
2'	115.2	114.5	131.2	115.3	116.6
3'	146.7	145.0	116.8	146.8	146.7
4'	149.7	147.9	161.2	149.7	149.7
5'	116.5	115.8	116.8	116.5	115.3
6'	123.0	123.6	131.2	123.1	123.2
7'	147.2	147.0	146.8	147.9	148.1
8'	114.9	116.9	115.0	114.4	114.2
9'	169.0	169.9	169.0	168.5	168.1
	CD_3OD	D_2O	CD_3OD	CD_3OD	CD_3OD

Tab. 7S. ^{1}H-NMR of L-threonic acid and γ-L-threonic acid lactone (in CD_3OD = δ 3.30 ppm, 50 MHz; M = multiplicity)

		L-threonic acid		γ-L-threonic acid lactone	
H	M	(ppm)	*J* (Hz)	(ppm)	*J* (Hz)
2	*d*	4.19	1.2	4.20	7.6
3	*ddd*	3.97	1.2; 6.0; 7.3	4.29	7.6; 6.8; 7.2
4a	*dd*	3.66	6.0; -11.0	4.41	6.8; -9.1
4b	*dd*	3.61	7.3; -11.0	3.93	7.2; -9.1
